# A phase Ib/II study of xentuzumab, an IGF-neutralising antibody, combined with exemestane and everolimus in hormone receptor-positive, HER2-negative locally advanced/metastatic breast cancer

**DOI:** 10.1186/s13058-020-01382-8

**Published:** 2021-01-15

**Authors:** Peter Schmid, Marie-Paule Sablin, Jonas Bergh, Seock-Ah Im, Yen-Shen Lu, Noelia Martínez, Patrick Neven, Keun Seok Lee, Serafín Morales, J. Alejandro Pérez-Fidalgo, Douglas Adamson, Anthony Gonçalves, Aleix Prat, Guy Jerusalem, Laura Schlieker, Rosa-Maria Espadero, Thomas Bogenrieder, Dennis Chin-Lun Huang, John Crown, Javier Cortés

**Affiliations:** 1 Centre for Experimental Cancer Medicine, Barts Cancer Institute, Queen Mary University of London, London, UK; 2 Department of Drug Development and Innovation, Institut Curie, Paris, France; 3 Department of Oncology-Pathology, Karolinska Institutet and Breast Cancer Centre, Cancer Theme, Karolinska University Hospital, Stockholm, Sweden; 4 Department of Internal Medicine, Seoul National University Hospital, Cancer Research Institute, Seoul National University College of Medicine, Seoul, South Korea; 5 Department of Oncology, National Taiwan University Hospital, Taipei, Taiwan; 6 Department of Oncology, Ramon y Cajal University Hospital, Madrid, Spain; 7 Department of Oncology, UZ Leuven, Campus Gasthuisberg, Leuven, Belgium; 8 Department of Internal Medicine, National Cancer Center, Goyang, South Korea; 9 Department of Medical Oncology, Hospital Universitario Arnau de Vilanova de Lleida, Lleida, Spain; 10 Medical Oncology Unit, Hospital Clinico Universitario Valencia, Biomedical Research Institute INCLIVA, CIBERONC, Valencia, Spain; 11 Department of Medical Oncology, Ninewells Hospital, Tayside Cancer Centre, Dundee, UK; 12 Department of Medical Oncology, Institut Paoli Calmettes, Aix-Marseille University, CRCM, CNRS, INSERM, Marseille, France; 13 Translational Genomics and Targeted Therapeutics in Solid Tumors, IDIBAPS, Hospital Clínic of Barcelona, Barcelona, Spain; 14 Department of Medical Oncology, Centre Hospitalier Universitaire de Liège, and Liège University, Liège, Belgium; 15 External statistician on behalf of Boehringer Ingelheim Pharma GmbH & Co. KG., Staburo GmbH & Co. KG., Munich, Germany; 16 Medical Department (Clinical Operations), Boehringer Ingelheim España S.A, Barcelona, Spain; 17 Medical Department, Boehringer Ingelheim, RCV, Vienna, Austria; 18 Present Address: Amal Therapeutics SA, Geneva, Switzerland; 19 Medical Department, Boehringer Ingelheim Taiwan Limited, Taipei, Taiwan; 20 Present Address: MSD Taiwan, Taipei, Taiwan; 21 Department of Medical Oncology, St Vincent’s University Hospital, Dublin, Ireland; 22 Breast Cancer Group, Vall d’Hebron Institute of Oncology (VHIO), Barcelona, Spain; 23 Department of Oncology, IOB Institute of Oncology, Quironsalud Group, Madrid and Barcelona, Spain

**Keywords:** Breast cancer, HER2-negative, Hormone receptor-positive, Insulin-like growth factor, Xentuzumab

## Abstract

**Background:**

Xentuzumab—a humanised IgG1 monoclonal antibody—binds IGF-1 and IGF-2, inhibiting their growth-promoting signalling and suppressing AKT activation by everolimus. This phase Ib/II exploratory trial evaluated xentuzumab plus everolimus and exemestane in hormone receptor-positive, locally advanced and/or metastatic breast cancer (LA/MBC).

**Methods:**

Patients with hormone receptor-positive/HER2-negative LA/MBC resistant to non-steroidal aromatase inhibitors were enrolled. Maximum tolerated dose (MTD) and recommended phase II dose (RP2D) of xentuzumab/everolimus/exemestane were determined in phase I (single-arm, dose-escalation). In phase II (open-label), patients were randomised 1:1 to the RP2D of xentuzumab/everolimus/exemestane or everolimus/exemestane alone. Randomisation was stratified by the presence of visceral metastases. Primary endpoint was progression-free survival (PFS).

**Results:**

MTD was determined as xentuzumab 1000 mg weekly plus everolimus 10 mg/day and exemestane 25 mg/day. A total of 140 patients were enrolled in phase II (70 to each arm). Further recruitment was stopped following an unfavourable benefit-risk assessment by the internal Data Monitoring Committee appointed by the sponsor. Xentuzumab was discontinued; patients could receive everolimus/exemestane if clinically indicated. Median PFS was 7.3 months (95% CI 3.3–not calculable) in the xentuzumab/everolimus/exemestane group and 5.6 months (3.7–9.1) in the everolimus/exemestane group (hazard ratio 0.97, 95% CI 0.57–1.65; *P* = 0.9057). In a pre-specified subgroup of patients without visceral metastases at screening, xentuzumab/everolimus/exemestane showed evidence of PFS benefit versus everolimus/exemestane (hazard ratio 0.21 [0.05–0.98]; *P* = 0.0293). Most common any-cause adverse events in phase II were diarrhoea (29 [41.4%] in the xentuzumab/everolimus/exemestane group versus 20 [29.0%] in the everolimus/exemestane group), mucosal inflammation (27 [38.6%] versus 21 [30.4%]), stomatitis (24 [34.3%] versus 24 [34.8%]), and asthenia (21 [30.0%] versus 24 [34.8%]).

**Conclusions:**

Addition of xentuzumab to everolimus/exemestane did not improve PFS in the overall population, leading to early discontinuation of the trial. Evidence of PFS benefit was observed in patients without visceral metastases when treated with xentuzumab/everolimus/exemestane, leading to initiation of the phase II XENERA™-1 trial (NCT03659136).

**Trial registration:**

ClinicalTrials.gov, NCT02123823. Prospectively registered, 8 March 2013.

**Supplementary Information:**

The online version contains supplementary material available at 10.1186/s13058-020-01382-8.

## Introduction

Standard treatment for postmenopausal women with hormone receptor-positive, human epidermal growth factor receptor (HER2)-negative advanced breast cancer is endocrine therapy, using an aromatase inhibitor (AI; e.g. letrozole, anastrozole [non-steroidal], or exemestane [steroidal]), tamoxifen, or fulvestrant [1]. Despite initial benefit, disease progression typically ensues due to acquired endocrine resistance. Additionally, patients may have primary resistance, rendering them unresponsive to endocrine therapy [2].

Strategies to prevent acquired endocrine resistance include inhibition of cell-cycle progression in breast cancer cells using cyclin-dependent kinase (CDK) inhibitors [2]. The addition of a CDK 4/6 inhibitor (e.g. palbociclib, ribociclib, and abemaciclib) to an AI in endocrine therapy-naïve or endocrine-pre-treated patients, or to fulvestrant in endocrine-pre-treated patients, improved progression-free survival (PFS) and overall survival (OS) [3, 4] and is recommended in current treatment guidelines [1].

Alternatively, treatment or reversal of endocrine resistance may be achieved by targeting molecular pathways that become activated during acquired resistance [2]. While activating *ESR1* mutations have emerged as a key mechanism in resistance to AIs (but not to fulvestrant) [5], adaptive signalling via the mammalian target of rapamycin (mTOR)/phosphoinositide 3-kinase (PI3K)/protein kinase B (AKT) pathway also plays an important role [2]. Everolimus, an mTOR inhibitor, is approved in combination with exemestane for advanced breast cancer with endocrine resistance, having demonstrated improved PFS versus exemestane alone in the BOLERO-2 trial [6, 7]; however, in contrast to some CDK4/6 inhibitors [3, 4], OS benefit was not observed. Additionally, everolimus was associated with a higher incidence of adverse events (AEs), such as stomatitis, fatigue, and hyperglycaemia. Notably, everolimus plus exemestane has shown similar efficacy irrespective of prior CDK4/6 inhibitor therapy [8].

Insulin-like growth factor (IGF) signalling can influence cancer progression and prognosis, and drives resistance to various anti-cancer treatments [9]. In breast cancer, regulatory feedback loops between the IGF axis and the mTOR/PI3K/AKT pathway may limit the efficacy of mTOR inhibitor/endocrine therapy combinations due to compensatory IGF ligand-driven signalling [2, 10]. Preclinical data also suggest that IGF signalling may have a key role in non-visceral disease, particularly bone and lymph node metastases development [11, 12].

Xentuzumab (BI 836845), a humanised IgG1 monoclonal antibody, binds IGF-1 and IGF-2 ligands with high affinity and potently neutralises their proliferative signalling [10]. Xentuzumab has shown preclinical activity across a range of cancer types [10]. Xentuzumab monotherapy was associated with mild-to-moderate AEs, most commonly gastrointestinal disorders, and preliminary anti-tumour activity in two phase I studies [13]. We hypothesised that combining xentuzumab with everolimus and exemestane would block the negative feedback between IGF and PI3K/AKT/mTOR signalling, thus enhancing sensitivity and/or overcoming endocrine resistance. This phase I/II study (NCT02123823) evaluated the efficacy and safety of xentuzumab plus everolimus and exemestane, versus exemestane and everolimus alone, in women with locally advanced or metastatic breast cancer (MBC).

## Materials and methods

### Study design

This study comprised a single-arm, dose-escalation phase I part to determine the maximum tolerated dose (MTD) and recommended phase II dose (RP2D) of xentuzumab in combination with everolimus and exemestane, and a two-arm, open-label, randomised, parallel-design phase II part to evaluate the anti-tumour activity of this triple combination versus everolimus and exemestane.

The phase I part included a 7-day run-in with everolimus and exemestane to achieve steady state prior to xentuzumab treatment. Dose escalation proceeded using a 3 + 3 design. The starting dose was everolimus 10 mg orally once daily (QD) and xentuzumab 750 mg intravenously once weekly. All cohorts received oral exemestane 25 mg QD. In the open-label phase II part, patients were randomised (1:1), stratified by visceral involvement (yes/no; where visceral refers to lung, liver, brain, pleural, and peritoneal metastasis), to everolimus 10 mg QD plus exemestane 25 mg QD, or xentuzumab plus everolimus (at the RP2D determined in phase I) and exemestane 25 mg QD. Proactive management of stomatitis was mandated, including use of mouthwash. Treatment continued in 28-day cycles until progression, intolerable AEs, or other reasons.

The study was approved by the Institutional Review Board or Independent Ethics Committee, respectively, with jurisdiction for the participating sites and was performed in accordance with the Declaration of Helsinki and International Conference on Harmonisation Good Clinical Practice. All patients received oral and written study information and provided written, informed consent. In the phase II part, an internal data monitoring committee (DMC)—comprised of an independent group of experts from the sponsor—was appointed to assess the benefit-risk balance of xentuzumab in combination with everolimus and exemestane (two planned timepoints: after approximately 30 and 45 PFS events). Following the first analysis, the DMC advised termination of the study based on a claimed negative benefit-risk balance. Patient recruitment was stopped on 28 October 2016, and xentuzumab was discontinued; however, patients could continue to receive everolimus and exemestane if clinically indicated.

### Patients

Full criteria are provided in Supplementary methods (see Additional file 1). Briefly, eligible patients were postmenopausal women aged ≥ 18 years, with histologically confirmed, locally advanced or MBC that was not amenable to curative therapy, and had recurred or progressed on/after the last line of systemic therapy for breast cancer. Patients were required to have hormone receptor-positive, HER2-negative tumours per local testing, and adequate archival tumour tissue. Patients had tumours that were resistant to non-steroidal AIs (defined as recurrence during/within 12 months after adjuvant treatment or progression during/within 1 month after treatment for locally advanced or MBC). Additional inclusion criteria included the following: life expectancy of ≥ 6 months, adequate organ function, a measurable lesion per Response Evaluation Criteria in Solid Tumors (RECIST) v.1.1 or bone lesions (lytic or mixed) in the absence of a measurable lesion, and Eastern Cooperative Oncology Group performance status ≤ 2. Exclusion criteria included the following: previous treatment with agents targeting the IGF, PI3K, AKT, or mTOR pathways; prior exemestane treatment; brain or central nervous system metastases; and > 2 previous lines of chemotherapy for locally advanced/MBC (> 1 previous line in phase II). There was no limit on lines of prior endocrine therapies, and prior CDK4/6 inhibitor treatment was permitted.

### Endpoints and assessments

The primary phase I endpoint was MTD, defined as the highest dose at which no more than one of six MTD-evaluable patients experienced a dose-limiting toxicity (DLT; Supplementary Table 1, Additional File 1) during the first treatment cycle (MTD evaluation period). The primary phase II endpoint was PFS per investigator review (time from the date of randomisation until the date of the first progression according to RECIST v.1.1, or death). Secondary endpoints (phase II) were time to progression (TTP), objective response (OR; complete response [CR] or partial response [PR] per RECIST v.1.1), time to OR, duration of OR, disease control (CR or PR, or stable disease or non-CR/non-progressive disease lasting ≥ 24 weeks), and duration of disease control. Further phase II endpoints included OS (duration from date of randomisation to the date of death). AEs were graded per the National Cancer Institute Common Terminology Criteria for Adverse Events (NCI CTCAE) v.4.03.

### Statistical analysis

In phase II, sample size was calculated based on a hazard ratio (HR) of 0.72, equating to a median PFS increase from 7.8 months for everolimus/exemestane to 10.8 months for the triple combination. It was calculated that 90 PFS events were required, with an estimated study duration of around 30 months. Based on these considerations, 150 patients were planned to be randomised. Following the DMC recommendation to terminate the study, recruitment was stopped and xentuzumab treatment discontinued. To allow a meaningful treatment group comparison, analysis of all RECIST endpoints was based on a data cut-off of 25 November 2016. Mandatory collection of OS data was also stopped after a protocol amendment.

No formal statistical testing was planned; all analyses were exploratory. Efficacy analysis (phase II) included all randomised patients; safety analyses included all treated patients. A Cox proportional hazards model stratified by visceral involvement (yes/no) was used to estimate the HR for PFS between treatment arms. Median PFS was calculated from Kaplan–Meier curves with 95% confidence intervals (CIs) using the Greenwood variance estimate. Exploratory *P* values were calculated by two-sided log-rank test. OR rates (ORRs) and disease control rates (DCRs) were compared based on the odds ratio resulting from logistic regression, adjusted for visceral involvement. Exploratory *P* values were calculated using the likelihood ratio test. Pre-specified subgroup analyses for PFS included visceral involvement, bone-only metastasis, age, and measurable disease at baseline. Median duration of follow-up was calculated via the reverse Kaplan–Meier method.

## Results

### Phase I

#### Patients and treatment

Twenty-four patients were treated in phase I (xentuzumab 750 mg + everolimus 10 mg + exemestane 25 mg [Xe750+Ev10+Ex25], *n* = 3; xentuzumab 1000 mg + everolimus 10 mg + exemestane 25 mg [Xe1000+Ev10+Ex25], *n* = 21; Supplementary Fig. 1 and Supplementary Table 2, Additional File 1).

#### MTD and DLTs

During the MTD evaluation period, 0/3 patients in the Xe750+Ev10+Ex25 group and 1/6 patients in the Xe1000+Ev10+Ex25 group had a DLT (grade 3 stomatitis). MTD was thus determined as Xe1000+Ev10+Ex25.

#### Safety

The overall safety profile in phase I is shown in Supplementary Table 3 (Additional File 1). The most common any-grade AEs were anaemia (66.7%), decreased appetite (58.3%), hyperglycaemia (58.3%), and mucosal inflammation (54.2%).

### Phase II

#### Patients and treatment

In the phase II part, 140 patients were randomised (70 to each treatment), and 139 were treated (one patient in the Ev10+Ex25 arm died and was not treated; Supplementary Fig. 1, Additional File 1). Baseline characteristics were generally well balanced between the two treatment arms (Table 1). Median (range) duration of any study treatment was 3.7 (0.3–30.0) months in the Xe1000+Ev10+Ex25 arm and 5.5 (0.6–32.3) months in the Ev10+Ex25 arm.

**Table 1:** Baseline characteristics: phase II part

	Xe1000+Ev10+Ex25 (***n*** = 70)	Ev10+Ex25 (***n*** = 70)	Total (***n*** = 140)
Median age, years (range)	58.5 (41–86)	62.0 (42–84)	60.0 (41–86)
ECOG PS, *n* (%)			
0	45 (64.3)	38 (54.3)	83 (59.5)
1	25 (35.7)	31 (44.3)	56 (40.0)
2	0	1 (1.4)	1 (0.7)
Median time since diagnosis, months (range)	63.4 (12.4–343.5)	63.3 (8.3–256.6)	63.3 (8.3–343.5)
Metastatic sites at screening, *n* (%)			
1	18 (25.7)	16 (22.9)	34 (24.3)
2	25 (35.7)	27 (38.6)	52 (37.1)
≥ 3	27 (38.6)	27 (38.6)	54 (38.6)
Visceral involvement, *n* (%)			
Yes	53 (75.7)	54 (77.1)	107 (76.4)
No	17 (24.3)	16 (22.9)	33 (23.6)
Bone metastases, *n* (%)	47 (67.1)	52 (74.3)	99 (70.7)
Lymph node metastases, *n* (%)	33 (47.1)	25 (35.7)	58 (41.4)
Prior hormone therapy, *n* (%)	68 (97.1)	69 (98.6)	137 (97.9)
Prior chemotherapy in metastatic setting, *n* (%)	17 (24.3)	18 (25.7)	35 (25.0)
Prior CDK 4/6 inhibitor treatment, *n* (%)	0	2 (2.9)	2 (1.4)

*CDK* cyclin-dependent kinase, *ECOG PS* Eastern Cooperative Oncology Group performance status

#### Efficacy

At the time of data cut-off for the RECIST endpoints, median duration of follow-up was 5.2 months (95% CI 3.5–5.5).

#### PFS

**Fig. 1:**
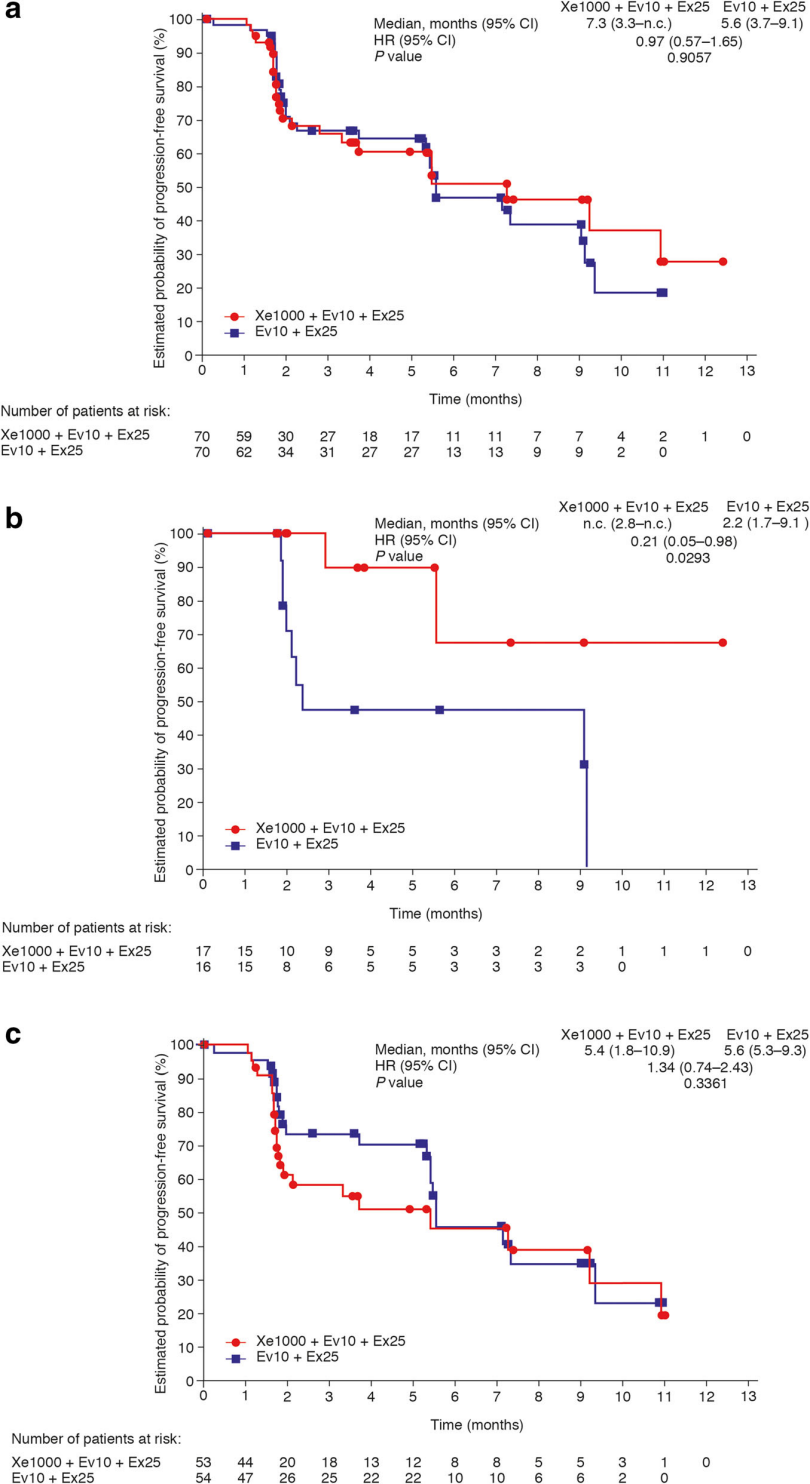
PFS in **a** all patients, **b** patients without visceral involvement, and **c** patients with visceral involvement. Data are from phase II part. CI, confidence interval; HR, hazard ratio; n.c., not calculable; PFS, progression-free survival

PFS was not significantly different between the Xe1000+Ev10+Ex25 and Ev10+Ex25 treatment arms (median [95% CI] PFS, 7.3 months [3.3–not calculable] versus 5.6 [3.7–9.1]; HR 0.97 [95% CI 0.57–1.65]; *P* = 0.9057; Fig. 1a).

#### Pre-specified subgroup analysis: visceral and non-visceral disease

**Fig. 2:**
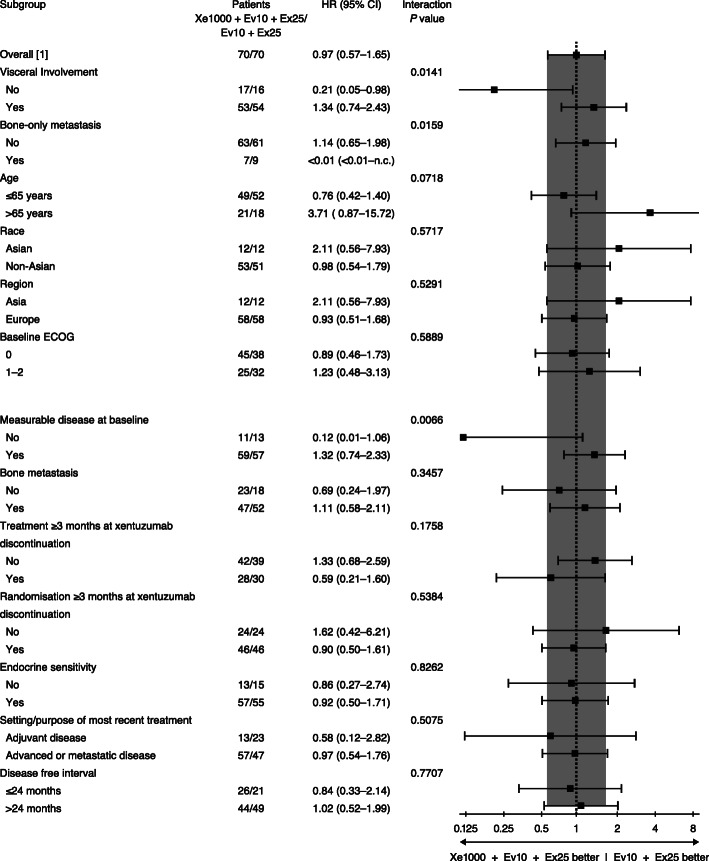
Subgroup analysis for PFS. Data are for phase II part; [1] overall results calculated as in the primary model analysis. ECOG, Eastern Cooperative Oncology Group; PFS, progression-free survival

In the pre-specified subgroup of patients without visceral metastases at screening, Xe1000+Ev10+Ex25 showed evidence of PFS benefit versus Ev10+Ex25 (two PFS events in 17 patients versus nine events in 16 patients; HR 0.21 [95% CI 0.05–0.98], *P* = 0.0293). Median PFS in those without visceral metastases was not calculable (95% CI 2.8–not calculable) for Xe1000+Ev10+Ex25 versus 2.2 months (95% CI 1.7–9.1) for Ev10+Ex25 (Fig. 1b). There was no evidence of PFS benefit in patients with visceral involvement (23 PFS events in 53 patients [Xe1000+Ev10+Ex25] versus 21 events in 54 patients [Ev10+Ex25]; HR 1.34 [95% CI 0.74–2.43], *P* = 0.3361). Median PFS in those with visceral metastases at screening was 5.4 months (95% CI 1.8–10.9) with Xe1000+Ev10+Ex25 versus 5.6 months (95% CI 5.3–9.3) with Ev10+Ex25 (Fig. 1c). The treatment by subgroup interaction *P* value [*P*_int_] was 0.0141. Evidence of PFS improvement was also observed in the nested “bone-only” metastasis subgroup (HR < 0.01 [< 0.01–not calculable]; *P* = 0.0942; *P*_int_ = 0.0159; Fig. 2).

#### Secondary and further endpoints

TTP was similar between treatment arms (HR 1.03 [95% CI 0.59–1.80]; *P* = 0.9146). ORRs and DCRs were also similar between the two treatment arms (Table 2). At the time of analysis, OS data were immature and inconsistently collected. Most patients (85.7%) had OS status censored at analysis. Overall, 20 patients had died (Xe1000+Ev10+Ex25, *n* = 9; Ev10+Ex25, *n* = 11; HR 0.79 [95% CI 0.33–1.93]).

**Table 2:** Best overall response: phase II part

	Xe1000+Ev10+Ex25 (***n*** = 70)	Ev10+Ex25 (***n*** = 70)
Best overall response		
CR	1 (1.4)	0
PR	4 (5.7)	7 (10.0)
Non-CR/non-PD, *n* (%)	10 (14.3)	8 (11.4)
Non-CR/non-PD ≥ 24 weeks	4 (5.7)	3 (4.3)
SD, *n* (%)	28 (40.0)	32 (45.7)
SD ≥ 24 weeks	4 (5.7)	7 (10.0)
PD, *n* (%)	16 (22.9)	14 (20.0)
Not evaluable, *n* (%)	11 (15.7)	9 (12.9)
OR, *n* (%)	5 (7.1)	7 (10.0)
Odds ratio (95% CI) [*P* value]^†^	0.70 (0.20–2.32) [*P =* 0.5598]	
Median time to OR, months (range)	3.7 (1.8–5.3)	1.8 (1.6–7.2)
Median duration of OR,^‡^ months (95% CI)	5.6 (NC–NC)	NC (1.8–NC)
Disease control, *n* (%)	13 (18.6)	17 (24.3)
Odds ratio (95% CI) [*P* value]^†^	0.70 (0.31–1.59) [*P =* 0.4008]	
Median duration of disease control,^‡^ months (95% CI)	NC (9.2–NC)	9.3 (9.0–NC)

*CI* confidence interval, *CR* complete response, *NC* not calculable, *OR* objective response, *PD* progressive disease, *PR* partial response, *SD* stable disease

^†^Odds ratio and *P* value are obtained from logistic regression model adjusted for visceral involvement at screening. An odds ratio > 1 favours Xe1000+Ev10+Ex25

^‡^Kaplan–Meier estimates

#### Safety

Following the DMC recommendation, xentuzumab treatment was discontinued; however, patients could continue to receive exemestane/everolimus. Safety analysis was therefore split according to AEs that occurred up to 9 December 2016 (i.e. the date of xentuzumab discontinuation [28 October 2016] plus the 42-day residual effect period; Table 3), and those occurring when patients were receiving only exemestane/everolimus on both treatment arms (10 December 2016 until 4 July 2018; Supplementary Table 4, Additional File 1).

**Table 3:** Safety profile and most common AEs occurring up to 9 December 2016: phase II part

Patients, ***n*** (%)	Xe1000+Ev10+Ex25 (***n*** = 70)		Ev10+Ex25 (***n*** = 69)	
**Any AE**	70 (100.0)		68 (98.6)	
**Any grade ≥ 3 AE**	39 (55.7)		31 (44.9)	
**Any TRAE**	66 (94.3)		66 (95.7)	
**Any grade ≥ 3 TRAE**	31 (44.3)		21 (30.4)	
**Any serious AE**	15 (21.4)		20 (29.0)	
**Most common any-cause AEs**	**Any grade**	**Grade ≥ 3**	**Any grade**	**Grade ≥ 3**
Diarrhoea	29 (41.4)	1 (1.4)	20 (29.0)	1 (1.4)
Mucosal inflammation	27 (38.6)	2 (2.9)	21 (30.4)	0
Stomatitis	24 (34.3)	4 (5.7)	24 (34.8)	3 (4.3)
Nausea	24 (34.3)	0	16 (23.2)	0
Rash	23 (32.9)	0	21 (30.4)	0
Cough	23 (32.9)	1 (1.4)	16 (23.2)	2 (2.9)
Asthenia	21 (30.0)	1 (1.4)	24 (34.8)	2 (2.9)
Fatigue	20 (28.6)	0	17 (24.6)	1 (1.4)
Neutropenia	20 (28.6)	8 (11.4)	11 (15.9)	2 (2.9)
Decreased appetite	18 (25.7)	1 (1.4)	21 (30.4)	0
Hyperglycaemia	18 (25.7)	4 (5.7)	15 (21.7)	3 (4.3)
Headache	17 (24.3)	0	9 (13.0)	0
Thrombocytopenia	17 (24.3)	4 (5.7)	10 (14.5)	1 (1.4)
ALT increased	16 (22.9)	3 (4.3)	10 (14.5)	1 (1.4)
Anaemia	15 (21.4)	2 (2.9)	11 (15.9)	2 (2.9)
Vomiting	14 (20.0)	2 (2.9)	13 (18.8)	0
AST increased	13 (18.6)	3 (4.3)	13 (18.8)	0
Epistaxis	13 (18.6)	0	9 (13.0)	1 (1.4)
Platelet count decreased	13 (18.6)	1 (1.4)	3 (4.3)	0
Constipation	13 (18.6)	1 (1.4)	6 (8.7)	0
Pruritus	13 (18.6)	0	12 (17.4)	0
Nasopharyngitis	12 (17.1)	0	6 (8.7)	0
Hypophosphataemia	11 (15.7)	6 (8.6)	8 (11.6)	2 (2.9)
Dysgeusia	11 (15.7)	0	10 (14.5)	0
Dyspnoea	9 (12.9)	1 (1.4)	17 (24.6)	4 (5.8)
Peripheral oedema	5 (7.1)	0	14 (20.3)	0
Arthralgia	4 (5.7)	0	11 (15.9)	0
Mouth ulceration	3 (4.3)	0	12 (17.4)	0

*AE* adverse event, *ALT* alanine aminotransferase, *AST* aspartate aminotransferase, *TRAE* treatment-related adverse event. Most common AEs are those of any causality (≥ 15% of patients in either arm)

Any-cause AEs reported more frequently with Xe1000+Ev10+Ex25 versus Ev10+Ex25 were abdominal pain (14.3% versus 4.3%), diarrhoea (41.4% versus 29.0%), headache (24.3% versus 13.0%), muscle spasms (11.4% versus 1.4%), nausea (34.3% versus 23.2%), neutropenia (28.6% versus 15.9%), and platelet count decreased (18.6% versus 4.3%). Infusion-related reactions with xentuzumab were rarely reported (2 [2.9%] patients; one grade 1 and one grade 2). Selected AEs associated with everolimus were examined using grouped terms (indicated by +). The incidence of stomatitis+ and non-infectious pneumonitis+ was similar between the two arms (74.3% versus 72.5% and 12.9% versus 18.8%, respectively). Neutropenia+ and thrombocytopenia+ were reported more frequently in the triple combination arm than the control arm (40.0% versus 27.5% [grade ≥ 3, 17.1% versus 7.2%] and 40.0% versus 17.7% [grade ≥ 3, 7.1% versus 1.4%], respectively).

Two patients in the Xe1000+Ev10+Ex25 arm died due to pneumonitis and liver injury, and two patients in the Ev10+Ex25 arm died due to progression of Burkitt’s lymphoma and acute kidney injury. Except for Burkitt’s lymphoma, these were considered related to treatment. The AE profile in patients without visceral involvement at screening was consistent with that in the overall trial population (data not shown).

Xentuzumab dose reduction was required in three patients in the Xe1000+Ev10+Ex25 arm. Everolimus dose reduction was required more frequently in the Xe1000+Ev10+Ex25 arm than the Ev10+Ex25 arm (32 [45.7%] versus 18 [26.1%] patients). The most frequent AE leading to dose reduction of everolimus in the Xe1000+Ev10+Ex25 arm was stomatitis (14 [20.0%] patients). However, discontinuations of everolimus due to AEs were numerically lower with Xe1000+Ev10+Ex25 than Ev10+Ex25 (9 [12.9%] versus 12 [17.4%] patients).

## Discussion

In phase I, the MTD of xentuzumab was 1000 mg weekly in combination with everolimus 10 mg QD and exemestane 25 mg QD, which was also determined as the RP2D. In the randomised phase II part, we hypothesised that addition of xentuzumab to everolimus/exemestane would overcome resistance to endocrine therapy; however, at a pre-specified interim analysis on 18 October 2016, the internal DMC recommended terminating the trial due to a lack of superior efficacy in the Xe1000+Ev10+Ex25 arm. Consequently, patient recruitment was stopped early and xentuzumab was discontinued. Patients continued to receive everolimus/exemestane provided they had not met discontinuation criteria.

Overall, the triple combination had a manageable and tolerable safety profile, which was remarkably similar to the safety profile of everolimus and exemestane alone. Frequency of key side effects associated with everolimus, such as pneumonitis, stomatitis/mucositis, and hyperglycaemia, was generally not increased by the addition of xentuzumab. In the phase II part, neutropenia and platelet count decreased were more common in the xentuzumab arm; however, this was not associated with increased infections.

While comparison of treatment arms was exploratory, there was no evidence of any difference in PFS between Xe1000+Ev10+Ex25 and Ev10+Ex25, and no relevant differences between treatment arms were observed regarding secondary or further efficacy endpoints. In line with the hypothesis that IGF may be important in bone and lymph node metastases, patients with non-visceral involvement were identified as a subgroup with potential benefit from treatment with Xe1000+Ev10+Ex25 versus Ev10+Ex25 (HR 0.21 [95% CI 0.05–0.98]). There was no evidence of a treatment effect for patients with visceral disease. Evidence of PFS improvement with Xe1000+Ev10+Ex25 versus control was also observed in the “bone-only” metastasis subgroup. Some caution should be taken with drawing any firm conclusions from the subgroup analyses given the number of subgroup analyses performed and the relatively low number of patients/events in some subgroups. Any comparisons of point estimates, such as medians, should also take into consideration corresponding 95% CIs.

The finding of benefit in the non-visceral subgroup is interesting, particularly as in BOLERO-2, the treatment effect of Ev10+Ex25 versus Ex25 was consistent in patients with and without visceral metastases [6]. However, fulvestrant has shown improved treatment effects in patients with non-visceral disease in the FIRST and FALCON trials (fulvestrant versus anastrozole). In these trials, which included endocrine treatment-naïve postmenopausal women with advanced breast cancer, the HRs for PFS were smaller for the non-visceral subgroup than the visceral subgroup (FALCON, 0.59 [95% CI 0.42–0.84] versus 0.99 [0.74–1.33]; FIRST, 0.58 [0.34–0.99] versus 0.82 [0.54–1.26]) [14]. Conversely, the PFS and OS HRs were similar in the non-visceral and visceral subgroups in CONFIRM (fulvestrant 500 versus 250 mg in patients with prior endocrine therapy; PFS, 0.72 [0.57–0.92] versus 0.86 [0.69–1.06]; OS, 0.78 [0.61–1.01] versus 0.83 [0.67–1.04]) [14].

The IGF axis is known to contribute to bone metastases, and bone-derived IGF has been shown to mediate crosstalk between bone and metastasized cancer cells [15]. Moreover, preclinical evidence suggests that inhibition of IGF-axis signalling can attenuate the development and progression of bone metastases in breast and prostate cancers [11]. Associations of elevated expression of IGF axis components with lymph node metastases have been identified in several cancer types, including breast cancer [12, 16, 17]. Interestingly, high IGF-1R expression appears to be associated with lower metastatic potential in breast cancer [18]. This may suggest that non-visceral disease retains IGF-1R signalling, while visceral metastases lose IGF-1R expression. These results are hypothesis-generating and warrant further examination of xentuzumab in patients with only non-visceral metastases.

## Conclusions

Overall, xentuzumab can be safely co-administered with everolimus and exemestane, but the addition of xentuzumab was not associated with improved PFS in the overall population of patients with hormone receptor-positive, HER2-negative locally advanced/MBC. Nonetheless, patients with no visceral involvement or bone-only metastases were identified as subgroups with potential benefit from treatment with Xe1000+Ev10+Ex25, which warrants further investigation. Xentuzumab is currently being investigated in combination with everolimus and exemestane in a double-blind, placebo-controlled, randomised phase II trial (XENERA™-1) in women with hormone receptor-positive/HER2-negative locally advanced/MBC and non-visceral disease (NCT03659136). The inclusion criteria of XENERA™-1 also reflect the changing landscape in hormone receptor-positive MBC. Given the time the current trial was conducted (enrolment between 2014 and 2016), there were very limited data on prior treatment with a CDK 4/6 inhibitor. Indeed, only two patients had received prior CDK4/6 inhibitors (Table 1). One patient received prior abemaciclib (January 2015 to July 2015) and one received prior palbociclib (April 2015 to June 2015). In the current trial, both were randomised to Ev10+Ex25 and had PFS of 5.4 and 1.5 months, respectively. Patients enrolled in the XENERA™-1 trial are permitted to have received one prior treatment line with a CDK 4/6 inhibitor, and randomisation will also be stratified based on this.

## Supplementary Information

**Additional file 1.** : Supplementary material. Supplementary methods, tables and figure

## Data Availability

To ensure independent interpretation of clinical study results, Boehringer Ingelheim grants all external authors access to all relevant material, including participant-level clinical study data, and relevant material as needed by them to fulfil their role and obligations as authors under the ICMJE criteria. Furthermore, clinical study documents (e.g. study report, study protocol, statistical analysis plan) and participant clinical study data are available to be shared after publication of the primary manuscript in a peer-reviewed journal and if regulatory activities are complete and other criteria met per the BI Policy on Transparency and Publication of Clinical Study Data: https://trials.boehringer-ingelheim.com/transparency_policy.html Prior to providing access, documents will be examined and, if necessary, redacted and the data will be de-identified, to protect the personal data of study participants and personnel, and to respect the boundaries of the informed consent of the study participants. Clinical Study Reports and Related Clinical Documents can be requested via this link: https://trials.boehringer-ingelheim.com/trial_results/clinical_submission_documents.html All such requests will be governed by a Document Sharing Agreement. Bona fide, qualified scientific and medical researchers may request access to de-identified, analysable participant clinical study data with corresponding documentation describing the structure and content of the datasets. Upon approval, and governed by a Data Sharing Agreement, data are shared in a secured data-access system for a limited period of 1 year, which may be extended upon request. Researchers should use https://trials.boehringer-ingelheim.com to request access to study data.

## Abbreviations

LA/MBC: Locally advanced and/or metastatic breast cancer
MTD: Maximum tolerated dose
RP2D: Recommended phase II dose
PFS: Progression-free survival
HER2: Human epidermal growth factor receptor
AI: Aromatase inhibitor
CDK: Cyclin-dependent kinase
OS: Overall survival
mTOR: Mammalian target of rapamycin
PI3K: Phosphoinositide 3-kinase
AKT: Protein kinase B
AEs: Adverse events
IGF: Insulin-like growth factor
MBC: Metastatic breast cancer
QD: Once daily
DMC: Data monitoring committee
RECIST: Response Evaluation Criteria in Solid Tumors
DLT: Dose-limiting toxicity
TTP: Time to progression
OR: Objective response
CR: Complete response
PR: Partial response
NCI CTCAE: National Cancer Institute Common Terminology Criteria for Adverse Events
HR: Hazard ratio
CIs: Confidence intervals
ORRs: Objective response rates
DCRs: Disease control rates
