# Supplementary material for: A phase Ib/II study of xentuzumab, an IGF-neutralising antibody, combined with exemestane and everolimus in hormone receptor-positive, HER2-negative locally advanced/metastatic breast cancer
Source: Breast Cancer Res. 2021 Jan 15;23:8. doi: 10.1186/s13058-020-01382-8 (PMC7811234; doi:10.1186/s13058-020-01382-8)

**Additional file 1**

**Supplementary Methods**

Patients with histologically-confirmed locally advanced or metastatic breast cancer not deemed amenable to curative surgery or curative radiation therapy, with tumours positive for oestrogen-receptor (ER) and/or progesterone receptor, could be included in this trial.

***Inclusion criteria***

1. Histologically-confirmed locally advanced or metastatic breast cancer (mBC) not deemed amenable to curative surgery or curative radiation therapy.

2. Tumours are positive for ER and/or progesterone receptor (PgR). Tumours had to be negative for HER2 per local laboratory testing. ER, PgR and HER2 status previously assessed by a local laboratory was acceptable.

3. Must have had adequate archival tumour tissue from surgery or biopsy. If multiple surgeries/biopsies were available for individual patients, the most recent and/or the most appropriate tissue material was requested.

4. Postmenopausal female patients aged ≥18 years old. Postmenopausal status was defined either by: a. Age ≥55 years and one year or more of amenorrhea; b. Age <55 years and one year or more of amenorrhea, in the absence of chemotherapy, tamoxifen, toremifene, or ovarian suppression, and FSH and oestradiol within postmenopausal ranges as per institution standard/normal practice; c. Surgical menopause with bilateral oophorectomy.

5. Objective evidence of recurrence or progressive disease on or after the last line of systemic therapy for breast cancer prior to study entry.

6. The patient was disease refractory to non-steroidal aromatase inhibitor (letrozole and/or anastrozole): defined as recurrence during or within 12 months after the end of adjuvant treatment or progression during or within 1 month after the end of aromatase inhibitor treatment for locally advanced or metastatic disease. Note: Letrozole or anastrozole did not have to be the most recent treatment. Prior anticancer therapy, e.g. tamoxifen, fulvestrant were allowed.

7. Patients must have: a. Measurable lesion according to RECIST version 1.1 or b. Bone lesions: lytic or mixed (lytic + sclerotic) in the absence of measurable lesion as defined above.

8. Eastern Cooperative Oncology Group performance score ≤2.

9. Life expectancy of ≥6 months in the opinion of the investigator.

10. Fasting plasma glucose <8.9 mmol/L (<160 mg/dL) and HbA1c <8.0%.

11. Adequate organ function, defined as all of the following: a. Absolute neutrophil count ≥1500/mm^3^; b. Platelet count ≥100,000/mm^3^; c. International Normalized Ratio ≤2.0; d. Serum creatinine ≤1.5 times upper limit of institutional normal (ULN); e. Total Bilirubin ≤1.5 times ULN (patients with Gilbert syndrome total bilirubin must be <4 times institutional ULN); f. Aspartate amino transferase (AST) and alanine aminotransferase (ALT) ≤ three times ULN (if related to liver metastases ≤ five times ULN); g. Fasting triglycerides ≤300 mg/dL or 3.42 mmol/L; h. Haemoglobin (Hgb) ≥9.0 g/dL

12. Recovered from any previous therapy related toxicity to ≤Grade 1 at study entry (except for stable sensory neuropathy ≤Grade 2 and alopecia).

13. Written informed consent that was consistent with ICH-GCP guidelines and local regulations.

***Exclusion criteria***

Phase Ib part:

1. Previous treatment with agents targeting the IGF pathway, phosphoinositide 3-kinase (PI3K) signaling pathway, protein kinase B (AKT), or mammalian target of rapamycin (mTOR) pathways (sirolimus, temsirolimus, etc).

2. Prior treatment with exemestane (except adjuvant exemestane stopped >12 months prior to start of study treatment as long as the patient did not recur during or within 12 months after the end of adjuvant exemestane).

3. Known hypersensitivity to monoclonal antibody, mTOR inhibitors (e.g. sirolimus), or to the excipients of any study drugs.

4. Ovarian suppression by ovarian radiation or treatment with a luteinizing hormone-releasing hormone (LH-RH) agonist (goserelin acetate or leuprolide acetate).

5. Less than one week after receiving immunisation with attenuated live vaccines prior to study treatment.

6. Radiotherapy within 4 weeks prior to the start of study treatment, except in case of localized radiotherapy for analgesic purpose or for lytic lesions at risk of fracture which could then be completed within two weeks prior to study treatment.

7. Chemotherapy, biological therapy (other than bevacizumab), immunotherapy or investigational agents within 5 half-lives of the drug or within two weeks prior to the start of study treatment, whichever was longer; bevacizumab treatment within 4 weeks prior to start of study treatment (this criterion concerns anti-cancer therapy only).

8. Hormonal treatment for breast cancer within 2 weeks prior to start of study treatment.

9. Major surgery, in the judgement of the investigator, within 4 weeks before starting study treatment or scheduled for surgery during the projected course of the study.

10. Patients receiving concomitant immunosuppressive agents or chronic corticosteroids use except in cases outlined as follows: a. Topical applications (e.g. rash), inhaled sprays (e.g. obstructive airways diseases), eye drops or local injections (e.g. intra-articular) were allowed; b. Patients on stable low dose of corticosteroids for at least two weeks before study entry were allowed

11. Chronic hepatitis B infection (defined as presence of HBsAg and/ or HBV-DNA), chronic hepatitis C infection (defined as presence of anti-HCV Ab and/or HCV-RNA) and/or known HIV carrier.

12. QTcF prolongation >470 ms or QT prolongation deemed clinically relevant by the investigator (e.g. congenital long QT syndrome). The QTcF was calculated as the mean of the 3 ECGs taken at screening.

13. Disease that was considered by the investigator to be rapidly progressing or life threatening such as extensive symptomatic visceral disease including hepatic involvement and pulmonary lymphangitic spread of tumour (subjects who were intended for urgent chemotherapy).

14. History or current presence of brain or other CNS metastases.

15. Bilateral diffuse lymphangitic carcinomatosis (in lung).

16. Hypokalemia of Grade >1.

17. History of another primary malignancy within 5 years, with the exception of adequately treated in-situ carcinoma of the cervix, uteri, basal or squamous cell carcinoma or non-melanomatous skin cancer.

18. Family history of long QT syndrome.

19. Any concomitant serious illness or organ system dysfunction which in the opinion of the investigator would either compromise patient safety or interfere with the evaluation of the safety and anti-tumour activity of the test drug(s) such as: a) History or presence of clinically relevant cardiovascular abnormalities such as uncontrolled hypertension, congestive heart failure, New York Heart Association (NYHA) functional classification of 3 or 4, unstable angina or poorly controlled arrhythmia, including any type of atrial fibrillation. Myocardial infarction within 6 month prior to the study entry. b) Impairment of gastrointestinal function or had gastrointestinal disease that may significantly alter the absorption of study drugs (e.g. ulcerative disease, uncontrolled nausea, vomiting, diarrhoea, malabsorption syndrome), or any GI disorders of Grade >1. c) Active skin, mucosa, or ocular disorders of Grade > 1. d) Significant symptomatic deterioration of lung function. If clinically indicated, pulmonary function tests including measures of predicted lung volumes, DLco, O2 saturation at rest on room air were to be considered to exclude restrictive pulmonary disease, pneumonitis or pulmonary infiltrates.

20. Patients being treated with drugs recognized as being strong or moderate CYP3A4 and/or P-glycoprotein (PgP) inhibitors and/or strong CYP3A4 inducers within 2 weeks (or use of amiodarone within 6 months) prior to study entry.

21. Patients unwilling or unable to comply with study and follow-up procedures in the opinion of the investigator.

22. Patients received more than two lines of chemotherapy for locally advanced or metastatic breast cancer. Note: A chemotherapy line in advanced/metastatic disease is an anticancer regimen(s) that contains at least 1 cytotoxic chemotherapy agent and given for 21 days or longer. If a cytotoxic chemotherapy regimen was discontinued for a reason other than disease progression and lasted less than 21 days, then this regimen did not count as a "prior line of chemotherapy". Locally advanced/metastatic disease was to be understood as not amenable for curative therapy/surgery. Therefore chemotherapy agents in the adjuvant/neoadjuvant setting should not be considered here.

Phase II Part:

Exclusion criteria for the Phase II part were identical to the Phase Ib part except that exclusion criteria number 22 of the Phase Ib part was replaced with the following exclusion criteria: Patients received more than one line of chemotherapy for locally advanced or metastatic breast cancer

**Supplementary Table 1** Criteria for DLT in Phase I (AEs or laboratory abnormalities considered related to study drug)

| Grade 3 hyperglycaemia lasting > 48 hours or Grade 4 hyperglycaemia |
| --- |
| Grade ≥3 stomatitis (oral mucositis) despite appropriate supportive care |
| Grade ≥3 thrombocytopenia lasting ≥7 days or thrombocytopenia associated with active bleeding or requiring platelet transfusion |
| Grade ≥4 decreased platelet count |
| Grade ≥3 febrile neutropenia and/or documented infection with ANC <1.0x10^9^/L |
| Grade 4 decreased neutrophil count lasting ≥7 days |
| AST or ALT >5x ULN (for baseline AST/ALT ≤ ULN) or > baseline value + 4x ULN (for baseline AST/ALT > ULN) |
| Grade ≥3 diarrhoea, nausea, or vomiting despite adequate supportive care |
| Grade ≥3 skin rash despite adequate supportive care measures |
| Grade ≥3 fatigue/asthenia lasting >7 days |
| Grade 3/4 hyperlipidaemia (total cholesterol > 400 mg/dL or triglycerides > 500 mg/dL) not improving despite appropriate treatment for 4 weeks |
| Any AE necessitating a 2-week treatment interruption |
| All other toxicities of Grade ≥3 (except alopecia, allergic reaction, infusion reaction and those mentioned above) |
| Any other drug-related toxicity considered significant enough to be qualified as  DLT in the opinion of the investigators |

AE, adverse event; ALT, alanine aminotransferase; ANC, absolute neutrophil count; AST, aspartate aminotransferase; DLT, dose-limiting toxicity; ULN, upper limit of normal

**Supplementary Table 2** Baseline characteristics: Phase I part

|  | **Xe750 + Ev10 + Ex25**  **(n=3)** | **Xe1000 + Ev10 + Ex25**  **(n=21)** | **Total**  **(n=24)** |
| --- | --- | --- | --- |
| Median age, years (range) | 59 (51–67) | 65 (49–80) | 65 (49–80) |
| ECOG PS, n (%) |  |  |  |
| 0 | 1 (33.3) | 13 (61.9) | 14 (58.3) |
| 1 | 2 (66.7) | 8 (38.1) | 10 (41.7) |
| Median time since diagnosis, months (range) | 29.6 (25.1–30.6) | 78.7 (16.6–382.9) | 75.3 (16.6–382.9) |
| Metastatic sites at screening, n (%) |  |  |  |
| 1 | 3 (100.0) | 4 (19.0) | 7 (29.2) |
| 2 | 0 | 11 (52.4) | 11 (45.8) |
| ≥3 | 0 | 6 (28.6) | 6 (25.0) |
| Visceral involvement, n (%) |  |  |  |
| Yes | 1 (33.3) | 18 (85.7) | 19 (79.2) |
| No | 2 (66.7) | 3 (14.3) | 5 (20.8) |
| Bone metastases, n (%) | 1 (33.3) | 19 (90.5) | 20 (83.3) |
| Lymph node metastases, n (%) | 0 | 5 (23.8) | 5 (20.8) |
| Prior hormone therapy, n (%) | 3 (100.0) | 21 (100.0) | 24 (100.0) |
| Prior chemotherapy in metastatic setting, n (%) | 1 (33.3) | 5 (23.8) | 6 (25.0) |

ECOG PS, Eastern Cooperative Oncology Group performance status

**Supplementary Table 3** Overall safety profile and most common AEs of any causality (≥30% of patients overall): Phase I part

| **Patients, n (%)** | **Xe750 + Ev10 + Ex 25**  **(n=3)** | | **Xe1000 + Ev10 + Ex25 (n=21)** | | **Total**  **(n=24)** | |
| --- | --- | --- | --- | --- | --- | --- |
| **Any AE** | 3 (100.0) | | 21 (100.0) | | 24 (100.0) | |
| **Any Grade ≥3 AE** | 2 (66.7) | | 13 (61.9) | | 15 (62.5) | |
| **Any TRAE** | 3 (100.0) | | 21 (100.0) | | 24 (100.0) | |
| **Any Grade ≥3 TRAE** | 1 (33.3) | | 13 (61.9) | | 14 (58.3) | |
| **Any serious AE** | 3 (100.0) | | 10 (47.6) | | 13 (54.2) | |
| **AE leading to xentuzumab dose reduction** | 0 | | 0 | | 0 | |
| **AE leading to everolimus dose reduction** | 0 | | 17 (81.0) | | 17 (70.8) | |
| **AE leading to exemestane dose reduction** | 0 | | 0 | | 0 | |
| **AE leading to xentuzumab discontinuation** | 3 (100.0) | | 4 (19.0) | | 7 (29.2) | |
| **AE leading to everolimus discontinuation** | 3 (100.0) | | 4 (19.0) | | 7 (29.2) | |
| **AE leading to exemestane discontinuation** | 3 (100.0) | | 4 (19.0) | | 7 (29.2) | |
| **Most common any-cause AEs** | **Any grade** | **Grade ≥3** | **Any grade** | **Grade ≥3** | **Any grade** | **Grade ≥3** |
| Anaemia | 3 (100.0) | 1 (33.3) | 13 (61.9) | 2 (9.5) | 16 (66.7) | 3 (12.5) |
| Hyperglycaemia | 3 (100.0) | 1 (33.3) | 11 (52.4) | 1 (4.8) | 14 (58.3) | 2 (8.3) |
| Decreased appetite | 1 (33.3) | 0 | 13 (61.9) | 0 | 14 (58.3) | 0 |
| Mucosal inflammation | 3 (100.0) | 0 | 10 (47.6) | 0 | 13 (54.2) | 0 |
| Weight decreased | 1 (33.3) | 0 | 11 (54.2) | 2 (9.5) | 12 (50.0) | 2 (8.3) |
| Asthenia | 3 (100.0) | 0 | 9 (42.9) | 0 | 12 (50.0) | 0 |
| Diarrhoea | 0 | 0 | 12 (57.1) | 0 | 12 (50.0) | 0 |
| Dysgeusia | 1 (33.3) | 0 | 9 (42.9) | 0 | 10 (41.7) | 0 |
| Nausea | 1 (33.3) | 0 | 9 (42.9) | 0 | 10 (41.7) | 0 |
| Pneumonitis | 1 (33.3) | 0 | 9 (42.9) | 0 | 10 (41.7) | 0 |
| Cough | 1 (33.3) | 0 | 9 (42.9) | 0 | 10 (41.7) | 0 |
| Blood creatine phosphokinase increased | 2 (66.7) | 0 | 7 (33.3) | 0 | 9 (37.5) | 0 |
| Hypertension | 0 | 0 | 9 (42.9) | 4 (19.0) | 9 (37.5) | 4 (16.7) |
| Epistaxis | 0 | 0 | 9 (42.9) | 0 | 9 (37.5) | 0 |
| Fatigue | 0 | 0 | 9 (42.9) | 0 | 9 (37.5) | 0 |
| Stomatitis | 0 | 0 | 8 (38.1) | 1 (4.8) | 8 (33.3) | 1 (4.2) |
| Vomiting | 1 (33.3) | 0 | 7 (33.3) | 0 | 8 (33.3) | 0 |
| Hypophosphatemia | 1 (33.3) | 0 | 7 (33.3) | 6 (28.6) | 8 (33.3) | 6 (25.0) |
| Neutropenia | 1 (33.3) | 1 (33.3) | 7 (33.3) | 0 | 8 (33.3) | 1 (4.2) |
| Rash | 2 (66.7) | 0 | 6 (28.6) | 0 | 8 (33.3) | 0 |
| Malignant neoplasm progression | 3 (100.0) | 0 | 5 (23.8) | 1 (4.8) | 8 (33.3) | 1 (4.2) |

AE, adverse event; TRAE, treatment-related adverse event

**Supplementary Table 4** Overall safety profile and most common AEs of any causality (≥15% of patients in either arm) with onset after 09 December, 2016: Phase II part

| **Patients, n (%)** | **Xe1000 + Ev10 + Ex25^†^**  **(n=41)** | | **Ev10 + Ex25^†^ (n=36)** | | **Total**  **(n=77)** | |
| --- | --- | --- | --- | --- | --- | --- |
| **Any AE** | 22 (53.7) | | 29 (80.6) | | 51 (66.2) | |
| **Any Grade ≥3 AE** | 6 (14.6) | | 10 (27.8) | | 16 (20.8) | |
| **Any TRAE** | 19 (46.3) | | 26 (72.2) | | 45 (58.4) | |
| **Any Grade ≥3 TRAE** | 2 (4.9) | | 5 (13.9) | | 7 (9.1) | |
| **Any serious AE** | 5 (12.2) | | 12 (33.3) | | 17 (22.1) | |
| **AE leading to everolimus dose reduction** | 2 (4.9) | | 4 (11.1) | | 6 (7.8) | |
| **AE leading to exemestane dose reduction** | 0 | | 0 | | 0 | |
| **AE leading to everolimus discontinuation** | 0 | | 4 (11.1) | | 4 (5.2) | |
| **AE leading to exemestane discontinuation** | 0 | | 0 | | 0 | |
| **Most common any-cause AEs** | **Any grade** | **Grade ≥3** | **Any grade** | **Grade ≥3** | **Any grade** | **Grade ≥3** |
| Anaemia | 4 (9.8) | 0 | 6 (16.7) | 2 (5.6) | 10 (13.0) | 2 (2.6) |
| Diarrhoea | 7 (17.1) | 0 | 5 (13.9) | 0 | 12 (15.6) | 0 |
| Asthenia | 7 (17.1) | 0 | 5 (13.9) | 0 | 12 (15.6) | 0 |
| Nasopharyngitis | 1 (2.4) | 0 | 6 (16.7) | 0 | 7 (9.1) | 0 |
| Weight decreased | 1 (2.4) | 0 | 6 (16.7) | 1 (2.8) | 7 (9.1) | 1 (1.3) |
| Arthralgia | 6 (14.6) | 0 | 7 (19.4) | 0 | 13 (16.9) | 0 |
| Back pain | 3 (7.3) | 0 | 6 (16.7) | 0 | 9 (11.7) | 0 |

^†^Randomised treatment groups: all patients in either group received only everolimus + exemestane after 28^th^ October, 2016

AE, adverse event; TRAE, treatment-related adverse event

**Supplementary Figure 1.** Patient disposition


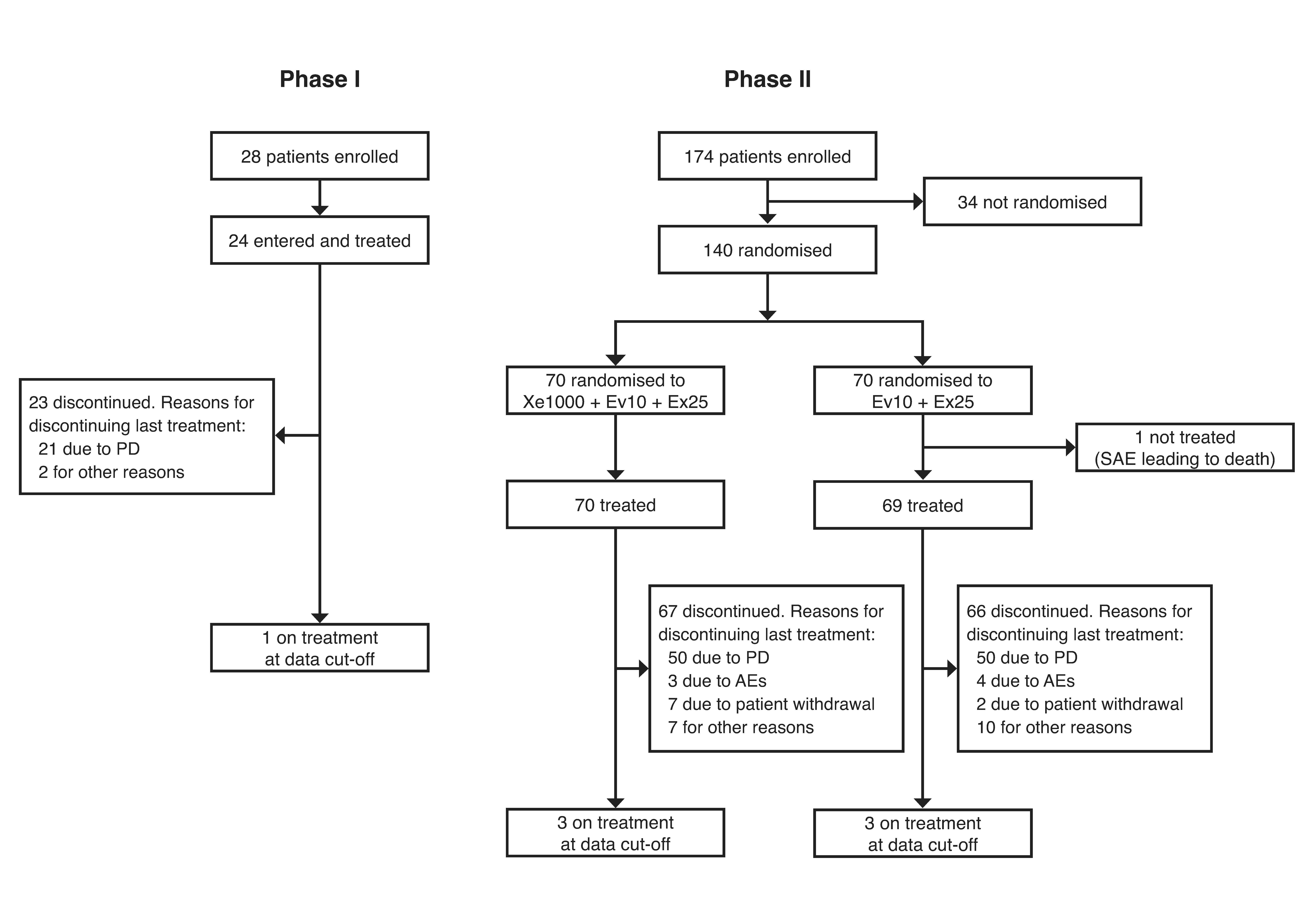

Supplement: Supplementary file 1 — Additional file 1. : Supplementary material. Supplementary methods, tables and figure [file 13058_2020_1382_MOESM1_ESM.docx]
